# The Relationship Between the Status of Unnecessary Accommodations Being Made to Unconfirmed Food Allergy Students and the Presence or Absence of a Doctor’s Diagnosis

**DOI:** 10.3390/children2020228

**Published:** 2015-06-01

**Authors:** Yurika Ganaha, Minoru Kobayashi, Yonathan Asikin, Taichi Gushiken, Sumie Shinjo

**Affiliations:** 1 Faculty of Education, University of the Ryukyus, 1 Senbaru, Nishihara, Okinawa 903-0213, Japan; 2 Center for Educational Career Enhancement, Kyoto University of Education, 1 Fukakusa-Fujinomori-cho, Fushimi-ku, Kyoto 612-8522, Japan; E-Mail: mkoba98@kyokyo-u.ac.jp; 3 Faculty of Life Sciences, Swiss German University, Edu Town BSD City, Kav. II.1, Tangerang 15339, Indonesia; E-Mail: yonathan.asikin@sgu.ac.id or yonathan.asikin@gmail.com; 4 Miyakojima Municipal Minami Elementary School, 1068 Shimozato, Hirara, Miyakojima, Okinawa 906-0013, Japan; E-Mail: nankuru68@hotmail.co.jp; 5 Okinawa Dietetic Association, 2-23-1 Takushi, Urasoe, Okinawa 901-2112, Japan; E-Mail: shinjots@beach.ocn.ne.jp

**Keywords:** children food allergies, school lunch, doctor’s diagnosis, unnecessary accommodations, school food services, nutrition and diet

## Abstract

The present study investigated the current state of unnecessary children food allergy accommodation and the medical efforts to confirm the existence of food allergies in school lunch service kitchens in Okinawa, Japan, including kitchens accommodating food allergy students by requiring medical documentation at the start and during provisions being made (Double Diagnosis), requiring medical documentation at the start only (Single Diagnosis), and with no medical documentation (Non-Diagnosis). Unnecessary accommodations are being made to unconfirmed food allergy students, wherein the more medical consultation was required, the lower the food allergy incident rate was and the more food allergens were diagnosed (Non-Diagnosis > Single Diagnosis > Double Diagnosis). This study suggests the possibility that unconfirmed food allergy students may be receiving unnecessary food allergy accommodations per school lunches, and the number of unnecessary food allergy provisions being made could be reduced by requiring medical documentation at the start and during these provisions.

## 1. Introduction

In 1954, Japan enacted the School Lunch Act aiming to provide every child with balanced nutrition while improving physical health and strength, from which point school lunches became a serious undertaking. According to the statistics published in 2012 by the Japanese Ministry of Education, Culture, Sports, Science and Technology [1], among the 33,386 national and public elementary, junior high, special needs, and night high schools, 30,295 (90.7%) of these schools provided students with a complete school lunch, which includes a main dish, side dish, and milk, which means that 9,379,774 or 88% of students are eating these school lunches. The age range of the children who attend elementary and junior schools is 6–15 years. As for the ratio between school lunch service kitchens, 42.6% of school lunches are prepared in kitchens and only provide lunch to that school (Single Cooking Place), while 55% of school lunches are prepared on sites not located within school grounds; these facilities (Combination Cooking Place) make and deliver lunches to multiple schools. Most of these facilities have one or more school nutritionists on site.

The school lunch menu is created by the on-site school nutritionist at each school lunch service kitchen in alignment with the national nutrition standards and food chart designated by the government, while at the same time prepared to be appetizing and full of variety. Each service kitchen complies with this menu and cooks meals for thousands of students; therefore, all students eat the same meal according to each school lunch service kitchen. However, because food allergies have become increasingly prevalent in Japan in recent years, in order to support these students with food allergies, it is being promoted that known food allergens be removed from school lunches so students with food allergies can be provided with safe school lunches. According to the research conducted in 2005 of all of the school lunch service kitchens in Japan, 63.2% are providing meals cooked without food allergens (Elimination Diet), and 26.2% of them are providing meals cooked without food allergens and adding alternative ingredients (Commutation Diet) [2].

Regardless of the country, children spend a good portion of their day living communally in a school environment, and in Japan, for example, it is estimated that the likelihood of children consuming food prepared in large quantities increases due to this environment. In many cases, the risk of a child developing a reaction due to food allergies increases significantly because the food allergen causing the reaction differs from child to child. In a study conducted in the United States involving 4586 children with peanut and other nut allergies, 750 of these participants developed food allergy symptoms at school or daycare [3]. Likewise, a survey taken in the United States reported that 18% of children have had an allergic reaction to food one or more times at school or preschool within two years [4]. In a national survey conducted in Japan during 2002–2004, there were 637 reported cases of students having an allergic due to school lunches [5]. Therefore, necessary accommodations for students with food allergies are not only necessary but urgent; many countries are taking initiative to implement the necessary provisions for these students [6,7,8,9,10].

An allergen-specific immunoglobulin E (IgE) blood test is typically used to diagnose food allergies; however, patients whose specific IgE antibody levels are positive sometimes exhibit no symptoms, while patients whose levels are negative can still have food allergy symptoms [11,12,13] and in the same way, positive skin tests may also yield no food allergy symptoms [14]. Due to this, the oral food challenge is reported to be the most reliable test in diagnosing food allergies [15]. Other research reported that the 27%–28% prevalence rate reported from questionnaires dropped to 2% when administering the oral food challenge instead [16]. However, the oral food challenge is a relatively new method of testing in Japan and was only added into health insurance coverage in 2006, and there are currently only 212 medical establishments within Japan which are allowed to give the oral food challenge and there are prefectures within Japan that do not have such a medical institution [17]. Consequently, there is not enough availability of oral food challenges, which are the most reliable in diagnosing food allergies, in contrast to the demand.

Since the Food Allergy Clinical Guidelines was first published in 2005, diagnosing food allergies has become a standard practice in Japan [18]. Because the implementation of blood tests and oral food tests to diagnose food allergies is not a requirement, the present situation is such that diagnostic methods differ depending on the doctor. Generally, in most cases, the doctor conducts a simple interview with the patient on onset history and an allergen-specific IgE blood test and diagnoses everything that tested positive as a food allergy. However, there are cases where doctor’s readily give a diagnosis based solely on the specific IgE test, regardless of the fact that food intake does not induce symptoms or only by a guardians’ complaint per interview. Although previous studies have voiced a concern over these unconfirmed food allergy students [2,19], the current status of this situation is not known. Moreover, children who have undergone a strict elimination diet due to their food allergies had, compared to the average, significantly lower body height and weight as well as showed a deficiency in nutritional intake [20,21,22] and unnecessary removal of foods is thought to be an interference and even an obstruction for healthy growth and development during adolescence. In addition, providing students with an elimination diet requires the effort and finances of the school lunch serving kitchen [23]. Consequently, giving unnecessary accommodations to unconfirmed food allergy students is undesirable to the child as well as the school, and it is essential to prevent before it occurs.

The present study thus aimed to determine the extent of unnecessary accommodations being made by the school lunch serving kitchens in Okinawa, Japan, and what kind of examinations are being conducted at the start and for the continuation of these accommodations for students. The students in this study were divided into three groups (Double Diagnosis group, Single Diagnosis group, and Non-Diagnosis group) based upon students’ initiative to receive medical examination at the start and for the continuation of their food allergies. This study also aimed to examine the relationship between whether or not an effort was made to confirm a doctor’s diagnosis, and the characteristics of unconfirmed food allergy students.

## 2. Methodology

### 2.1. Participants

The survey was conducted on 28–29 July 2011 during a workshop for school lunch nutrition staff and administration where all 114 school lunch service kitchens (excluding one kitchen facility due to renovations) in Okinawa Prefecture, Japan, were surveyed as participants using anonymous, self-completed questionnaires. The school lunch nutrition administration from 114 facilities filled out the surveys were once facility completed one survey and, in cases of multiple administration per school, a representative was asked to fill out the survey for his or her kitchen.

By way of introduction, school nutritionists mainly work in the school lunch kitchens and are not present at the school at all times. As for the situation of children with food allergies, they can be screened through the school health diagnosis; teachers can also receive an appeal from the child or their guardian that the child has a food allergy. The school nurse tabulates this per school and reports this to the school lunch kitchen nutritionist through the school principal. In rare cases, the child’s guardian may go directly to the school lunch service kitchen and request food allergy accommodations.

### 2.2. Instruments

The questionnaires were mainly multiple choice and were collected at the end of both workshop days, in order to capture the overall situation awareness among school nutritionists. Uncollected questionnaires were asked to be mailed within one week of the date. The questionnaire was comprised of fifty-five questions, including fourteen items of focus of the study which include the following: the number of meals provided to students, food accommodation availability, the number of food allergy students receiving accommodations, the number of food allergens each student is allergic to, the presence or absence of having questioned the validity of food allergy and specific examples of these instances (written answers), information on screening students at the beginning of food allergy accommodations, and information on screening students during food allergy accommodations. All questions were reviewed and approved by certified nutritionists and nutrition instructors at the University of the Ryukyus.

### 2.3. Data Analysis

**Figure 1:**
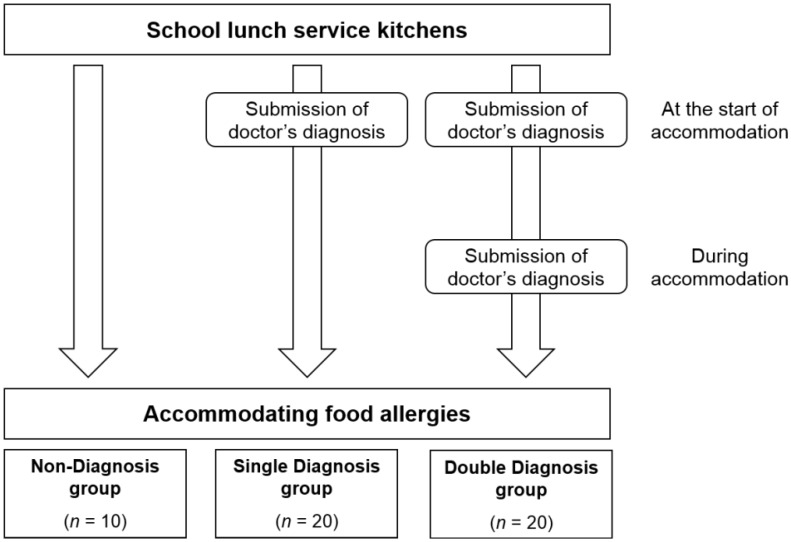
Outline of study groups (Double Diagnosis group, Single Diagnosis group, and Non-Diagnosis group) on school lunch service kitchens providing food allergy accommodations. * N = 50, the kitchen that did not respond as well as the kitchen only accommodating egg allergy students (two kitchens) have been excluded from the beginning so as not to produce a bias in the number of students.

Using school lunch service kitchens which provide food allergy accommodations as participants, this study divided up the kitchens into the following three groups: the Double Diagnosis group for school lunch service kitchens which require a doctor’s diagnosis at the beginning of and during food allergy accommodations, the Single Diagnosis group for kitchens which require a doctor’s diagnosis at the beginning of but not during food allergy accommodations in the, and the Non-Diagnosis group for kitchens which did not require a doctor’s diagnosis at the beginning of or during food allergy accommodations (Figure 1). Fisher’s exact test was conducted in order to examine whether or not there is a difference in the percentage of food allergy students within the three groups. Because the value among the three groups was statistically significant, the food allergy student ratio was compared by combining the groups into all possible groups of two and statistically comparing the differences between all two-group combinations. The two-sample test for equality of proportions compared the difference in ratio between the combinations of Double Diagnosis group × Single Diagnosis group, Double Diagnosis group × Non-Diagnosis group, Single Diagnosis group × Non-Diagnosis group. The school lunch serving kitchens were defined as “kitchens which require medical consultation” if they required any one of the following criteria: submission of medical documentation, submission of test results, submission of the school life management table, visits to a medical institution (no submission of medical submission required). Kitchens which did not meet this criteria were defined as “kitchens which do not require medical consultation.” The number of all students was determined through a cumulative total from each school lunch service kitchen and the number of students for which the kitchen provided meals. Afterward, a one-way analysis of variance was conducted in order to determine whether or not there is a difference in the number of food allergens to which the food allergy students are allergic among the three groups. Scheffe’s test was conducted for multiple comparisons with two-tailed test at significance level of 5% using SPSS Statistics 19 for Windows (SPSS Inc., Chicago, IL, USA) and Microsoft Office Excel 2007 (Microsoft Corp., Redmond, WA, USA).

### 2.4. Human Subjects Approval Statement

This study sought for the protection of privacy by ensuring the anonymity of each participant by using an anonymous, self-completed questionnaire, and the study protocol was approved by the Institutional Review Board on Epidemiological Research Ethics of the University of the Ryukyus.

## 3. Results

The response rate was 87% (100 out of 114 kitchens), 39% of which were simple cooking places and 61% were combination cooking places. Additionally, missing values from each item were excluded from all objects of analysis. Table 1 reveals the situation regarding food allergy accommodations in Okinawa, Japan. About 52% of school lunch service kitchens are accommodating students with food allergies while 41% of school lunch kitchens have not implemented accommodations into their school lunches despite their being a demand. Moreover, 7% of the kitchens are not providing accommodations as there are no students with a need for accommodations.

**Table 1:** The situation in school lunch service kitchens in accommodating food allergies (*N* = 100).

Description	*n* (%)
Accommodating food allergies	52 (52.0)
Not accommodating food allergies	41 (41.0)
There are no students with food allergies to accommodate	7 (7.0)

Regarding the presence or absence of having doubt when accommodating students with food allergies, it was revealed that 59.6% (*N* = 31) of school lunch service kitchens answered in the affirmative (Table 2). Examples of these instances contain medically inconsistent content, such as “The student has a dairy allergy but is able to eat yogurt,” and “The student is allergic to fish but can digest tuna and fish flakes,” and it is estimated that in 17 cases, the student has acquired tolerance to a specific food allergen. There were also 11 cases which infer excessive worrying by the guardians, such as, “Due to the parent’s assumptions, there is food which the student has not eaten since birth, and the parents insist the food be removed from school lunch as well,” and “The student’s medical records show an improvement in his food allergy; however, the parent’s insist dairy be removed from his school lunch.” There were also two cases questioning the legitimacy a doctor’s diagnosis, such as, “I question the credibility of the doctor’s diagnosis. A diagnosis has been written through interviews without a blood test.” These answers were obtained from 57.7% of those surveyed (30 school lunch services of the total 52 kitchens), for a total of 30 answers.

The requirement of medical documentation at the beginning of food allergy accommodations was generally implemented in the order of, “Submission of medical documentation (68.6%),” “Investigation of documentation (66.7%),” and “Investigation through interview with guardians (54.9%)” (Table 3). Next, an investigation was further conducted into whether or not schools were requiring examinations in order to determine if continuing food allergy accommodations was a necessity. About 56.9% of schools require medical examinations for continuing to receive accommodations, while the remaining 43.1% provide accommodations without requiring continuous documentation. In comparison to the percentages for the beginning, the overall implementation rate for continual medical documentation is low: “Submission of medical documentation (33.3%),” “Submission of test results (19.6%),” and, “Investigation of documentation (15.7%).” The investigation through food diaries allowed confirmation to be made on whether or not a specific food allergen was being ingested during family meals, and even though this information is powerful in speculating the presence or absence of having acquired tolerance to a food allergen, there was no implementation before or during food allergy accommodations.

**Table 2:** Specific examples of the presence of having questioned the validity of food allergies during accommodations *.

No.	Description	
Content contradicting what is medically known about food allergies and content estimating the acquisition of resistance towards food allergies:		
1	The student has a cheese allergy but is able to eat other dairy products.	
2	The students does not have a soy allergy but cannot eat roasted soybean flour due to food allergy symptoms occurring.	
3	We are accommodating a student with a shrimp allergy through an elimination diet, but the child complained and demanded, “Give me shrimp.” (There was medical documentation of a shellfish allergy)	
4	The student has a dairy allergy but is able to eat yogurt.	
5	There are many students who profess to having a food allergy but can tolerate a small amount.	
6	The student has a dairy allergy and is unable to drink milk but able to consume other processed dairy products.	
7	The student is allergic to fish but can digest tuna and fish flakes	
8	The student has a dairy allergy but is able to consume dairy when it is mixed with other ingredients	
9	The student has an egg allergy but can eat meatballs.	
10	A note from guardians indicated that their child has a dairy allergy but can have milk or cheese during school lunch as long as it is not two days in a row.	
11	The student has a shrimp allergy but is okay with picking out the shrimp himself. Is the extract from the shrimp okay?	
12	The student has a dairy allergy and eliminating milk from his diet but is still eating bread.	
13	The student cannot eat shrimp or crab but can digest the extract.	
14	The student cannot eat clams or squid but can eat clam chowder, meaning that he can probably eat it?	
15	The student supposedly has a cheese allergy but can eat gratin.	
16	The student has an egg allergy but is able to eat cake, *etc.* Another student has a soy allergy but is able to eat foods which includes soy products.	
17	The student has a soy allergy and is unable to eat soy itself but can digest tofu (soy sauce and miso are also okay). If he/she can eat tofu, cannot he/she eat soy itself?	
18	There was a complaint by parents whose child has a shrimp and crab allergy that their child had allergic reactions to the seasoning in the food; however, the seasoning we used was squid seasoning	
19	The parents decided that their child is allergic to a certain food because they are allergic to it themselves. The child has never had any tests done, so might there be a possibility he can eat it?	
20	The student’s medical records show an improvement in his food allergy; however, the parent’s insist dairy be removed from his school lunch.	
21	The guardians insist their child drink soy milk due to their policy (the student has been drinking soy milk since birth).	
22	The guardians are demanding their child be served tea instead of milk due to a dairy allergy; however, the student is eating foods with dairy ingredients.	
23	The requests for what the student cannot eat is different based on the doctor (blue fish), parents (raw fish) and him/herself (fish in general).	
24	Even though the student has not been diagnosed, because he had redness and itchiness after eating a certain food, the guardians decided it was probably a food allergy and he is now receiving accommodations.	
25	Due to the parent’s assumptions, there is food the student has not eaten since birth, and the parents insist the food be remove from school lunch as well.	
26	A student was diagnosed with a peanut allergy, but because the parents are worried, they requested all nuts be removed from the student’s diet.	
27	Because the student’s atopy became worse after eating a certain food allergen, an elimination diet was requested, but because the parents will not provide medical documentation, it may just be because of the student's likes/dislikes.	
28	Because the mother has been so serious since the student was young, she decided her child had a food allergy without any medical consultation.	
Questions concerning doctor’s diagnosis:		
29	I question the credibility of the doctor’s diagnosis.A diagnosis has been written through interviews without a blood test.	
30	The doctor’s diagnosis is not through an oral food test.	

* The total of school lunch services was 52 kitchens, wherein 31 kitchens (59.6%) answered “Yes” and 21 kitchens (40.4%) answered “No”, resulting 30 specific examples (57.7%).

**Table 3:** The contents of continuing medical examination before and during food allergy accommodations *.

Description	Number of School Lunch Service Kitchens Requiring Medical Examination, *n* (%)	
	At the start	During
Review of medical documentation	not applicable	29 (56.9)
Submission of medical documentation	35 (68.6)	17 (33.3)
Submission of test results	17 (33.3)	10 (19.6)
Submission of school life management table^#^	5 (9.8)	6 (11.8)
Only visits to a medical institution required (medical documentation not required)	4 (7.8)	2 (3.9)
Investigation through surveys created and conducted by the school lunch service kitchen	34 (66.7)	8 (15.7)
Investigation through food diary	0 (0.0)	0 (0.0)
Investigation through interview with guardians	28 (54.9)	7 (13.7)
Meeting with persons involved	19 (37.3)	6 (11.8)
Others	1 (2.0)	2 (3.9)

* Multiple answers allowed; N = 51, excluding one kitchen with missing data. #School Health Association, Japanese Ministry of Education, Culture, Sports, Science and Technology: Guideline 2008 for the Treatment of Allergic Diseases in Schools [24].

The students were divided into three groups based on requirements held by each school lunch service kitchen, which are the following: the Double Diagnosis group for kitchens requiring medical documentation at the start and during the provision of food allergy accommodations, the Single Diagnosis group for kitchens requiring medical documentation at the start but not during accommodations, and the Non-Diagnosis group for kitchens that required no medical documentation. This was done in order to compare the percentage of food allergy students receiving accommodations in each group. Excluding the items underlined previously in Table 3 (submission of medical documentation, submission of test results, submission of school life management table, only visits to a medical institution required) or those kitchens implementing more than one requirement, regardless, they were defined as either “kitchens which require medical consultation,” or “kitchens which do not require medical consultation.” Table 4 shows the comparison among the three groups. The occurrence rate of food allergy students was 3.55% in the Non-Diagnosis group, compared to 0.69% in the Single Diagnosis group and 0.40% in the Double Diagnosis group, showing the prevalence rate lowered the more each group sought out medical advice and a significant percentage difference of food allergy students among the three groups. Subsequently, Table 5 shows the comparison of the difference in ratios between two groups. In all of the groups, a substantial difference can be seen in the percentage of food allergy students: Double Diagnosis group < Single Diagnosis group, Double Diagnosis group < Non-Diagnosis group, Single Diagnosis group < Non-Diagnosis group.

**Table 4:** Comparison in the percentage of food allergy students in Double Diagnosis, Single Diagnosis, and Non-Diagnosis groups *.

Group	n *	Number of Students	Number of Students Receiving Food Allergy Accommodations (%)	*p*
Double Diagnosis	20	29048	117 (0.40)	
Single Diagnosis	20	17513	121 (0.69)	<.001
Non-Diagnosis	10	1887	67 (3.55)	

* N = 50, the kitchen that did not respond as well as the kitchen only accommodating egg allergy students have been excluded from the beginning so as not to produce a bias in the number of students.

**Table 5:** Differences in the percentage of food allergy students in two groups *.

Group Combination	Z	*p*
Double Diagnosis × Single Diagnosis	4.16	<.001
Double Diagnosis × Non-Diagnosis	17.08	<.001
Single Diagnosis × Non-Diagnosis	11.92	<.001

* *N* = 50, the kitchen that did not respond as well as the kitchen only accommodating egg allergy students have been excluded from the beginning so as not to produce a bias in the number of students.

Table 6 shows the comparison of the number of food allergens for students receiving food allergy accommodations in the Double Diagnosis group, Single Diagnosis group, and Non-Diagnosis groups. The average number of food allergens for food allergy students was 1.52 foods in the Non-Diagnosis group compared to 2.22 in the Single Diagnosis group and 2.42 in the Double Diagnosis group, showing an increase in the number of foods the more a group sought medical advice. The groups that showed significant differences were between Double Diagnosis group > Non-Diagnosis group and Single Diagnosis group > Non-Diagnosis group (*p* < .001).

**Table 6:** Comparison of the number of food allergens for students receiving food allergy accommodations in the Double Diagnosis, Single Diagnosis, and Non-Diagnosis groups.

Group	Number of Students Receiving Food Allergy Accommodations (%)	Food Allergens		*p*	Scheffe Comparison
		Average	Standard Deviation		
Double Diagnosis	108 (38.7)	2.42	1.63		Double Diagnosis > Non-Diagnosis Single Diagnosis > Non-Diagnosis
Single Diagnosis	112 (40.1)	2.22	1.59	<.001	
Non-Diagnosis	59 (21.1)	1.52	0.94		

## 4. Discussion

Although previous studies have expressed concern over food allergy accommodations being given to unconfirmed food allergy children, there has not been a sufficient examination of this problem [2,20]. The food allergen for food allergies differs depending upon age, but for the elementary and junior high students targeted in this study, the most prevalent food allergens were dairy (25.3%), eggs (25.0%), crustaceans (8.5%), and buckwheat (8.2%) [18]. In the present study, about 59.6% of school lunch service kitchens answered that they had questioned the validity of food allergies in food allergy students in Okinawa, Japan. Specific examples of this include such answers as, “The student has a dairy allergy but can eat yogurt,” which shows inconsistency with what is medically known, as well as examples that appear to show the student has acquired a tolerance to a specific food allergen. There were also examples which seem to imply parents’ excessive worrying, such as, “Due to the parent’s assumptions, there is food the student has not eaten since birth, and the parents insist the food be remove from school lunch as well.” The content from these examples show that the possibility of the presence of unconfirmed food allergy students may be extremely high. It is thus suggested that the current situation may be that unnecessary accommodations are being made to unconfirmed food allergy students per school lunches.

Regarding the medical examinations on children’s food allergies, there are school lunch service kitchens which require a doctor’s diagnosis through “Submission of medical documentation” and “Submission of test results,” and there are also school lunch service kitchens that give food allergy accommodations without requiring a doctor’s diagnosis, and it has become clear that the examination requirements and details differ greatly depending on each school lunch service kitchen. Once again, 43.1% of school lunch service kitchens have not implemented the practice of requiring examinations during the continuation of food allergy accommodations. This means that kitchens are continuing to accommodate students in the same way they have from the start, or the time of school admission, without confirming whether the students have acquired tolerance or without looking into any changes in their diseases. Even though there are kitchens which require examinations during the continuation of food allergy accommodations such as “Submission of medical documentation” and “Submission of test results” or such requirements that need a doctor’s diagnosis it is significantly lower than that of the beginning, at only 30%. From the above results, it has become apparent that 60% of school lunch service kitchens do not require a doctor’s diagnosis at the beginning or continuation of providing food allergy accommodations, and it is suggested that this is inadequate in order to prevent providing unnecessary accommodations to unconfirmed food allergy students. Unnecessary food allergy accommodations not only limits school activities, but it can also lead to the bullying of food allergy students and reduce the quality of life (QOL) of the student and family members [25].

In addition, because restricting diet can causes an adverse effect on students’ healthy growth and development, it is important that this be prevented if possible. Many common food allergens, such as eggs, dairy, soy, and meat, are high-quality protein foods necessary for the growth of developing children. It has been reported that those children who have undergone a strict elimination diet due to food allergies have lower height and weight than the average and have a tendency to be deficient in nutritional intake [20,21,22]. Furthermore, a previous study reported the cost and effort of introducing food allergy support in Japanese nurseries [23], which analyzed cooking time and ingredient expenses when two cooks prepared meals for a total of fifty meals, ten of which were commutation diet meals for food allergies. When assigning a value of 100% to the working time of a normal diet, cooking time would increase from 107 to 145% and ingredient expenses from 100 to 106% for commutation diet meals. Because this is a study conducted on nursery school lunches, it is not possible to use this as reference for school lunches; however, for the majority of school lunch service kitchens preparing over fifty meals per day, it is not hard to imagine that more time and expenses are being used by these kitchens.

Although “food allergy” is one phrase, it encompasses multiple food allergens and removal of these allergens is complex, while at the same time, food allergy support comes with a serious responsibility as the onset of food allergy symptoms has the potential of being life-threatening. As the amount of unnecessary food allergy accommodations increases, the risk of mistakes occurring heightens as the workload and complexity increases and ideally, the kitchen should be focusing on preparing the amount that is only necessary. Therefore, in order to prevent providing unnecessary food allergy provisions, students should be carefully examined at the beginning of and during food allergy accommodations and a doctor’s diagnosis is essential in order to objectively evaluate whether a student is a unconfirmed food allergy student or not. However, in the present study, the requirements surrounding examinations at the start and during food allergy accommodations varied significantly depending upon the school lunch service kitchen, and the requirement of submitting medical documentation provided by the doctor was insufficient. In March 2014 the Japanese Ministry of Education, Culture, Sports, Science and Technology distributed a notification statement to all of the schools and school lunch service kitchens in the nation based on the “Guideline 2008 for the Treatment of Allergic Diseases” issued in 2008 [24], urging that students submit a School Life Management Certificate for Allergic Diseases which has an entry column for a doctor’s diagnosis and signature. Although the present study has shed light on the fact that only 10% of school lunch service kitchens are utilizing the School Life Management Certificate for Allergic Diseases, the Ministry is hopeful and expectant that the notification will be used consistently from the present.

The relationship between whether or not an initiative was being taken to check a student’s doctor’s diagnosis and how this correlates to unconfirmed food allergy students, wherein the students were divided into three groups, Double Diagnosis group, Single Diagnosis group, and Non-diagnosis group, based on the whether or not a doctor’s diagnosis per an examination at the beginning and during food allergy accommodations was required. The number of food allergy students decreased in the order of Non-Diagnosis group (3.55%) > Single Diagnosis group (0.69%) > Double Diagnosis group (0.40%). Many previous studies have reported that children acquire tolerance as they develop, and although there is a discrepancy depending on the food allergen, 30–80% of food allergies heal naturally [26,27,28,29]. The present study revealed that when there are more opportunities to seek medical examination and a doctor’s diagnosis at both the beginning and during accommodations such as the Double Diagnosis group, it can be presumed to result in students visiting medical institutions and recognizing their acquired tolerance or excessively worried guardians being deterred from requesting food allergy accommodations for their child, ultimately omitting unconfirmed food allergy student and reducing the percentage of true food allergy students. From the fact that the ratio of food allergy students in the Double Diagnosis group was significantly lower than in the Single Diagnosis group, it can be suggested that, by requiring students to not only visit a medical institution at the beginning but also during the continuation of food allergy accommodations and mandating the submission of medical documentation and test results, there is a possibility to prevent unnecessary food allergy accommodations being given to unconfirmed food allergy students.

On the other hand, the average number of food allergens for food allergy students increased in the order of Non-Diagnosis group (1.52 foods) < Single Diagnosis group (2.22 foods) < Double Diagnosis group (2.42 foods). It is often the case with food allergies that tolerance is acquired as one develops, decreasing the number of foods allergens [26,27,28,29]. Therefore, although the number of food allergens were expected to decrease with an increase in doctor’s diagnosis, the results went against this expectation. The number of food allergens might have increased due to receiving excessive diagnosis by a nonspecialist for it is more common for food allergies to be diagnosed by a physician or pediatrician rather than a specialist along with the publication of Food Allergy Clinical Guidelines in 2005 [18]. This effect is expressed as “Non-specialists are diagnosing food allergies”, but whether or not physicians and pediatricians are diagnosing based on the guidelines are unknown and there are currently no reports to reveal this information. Specific IgE tests can result in positive specific IgE antibody level without the patient exhibiting food allergy symptoms and vice versa [11,12,13] and skin tests also can come out positive without symptoms as well [14] and reports have been made on the difficulty of diagnosing food allergies and excessive diagnoses by nonspecialists [30,31]. The present study has considered the possibility of students receiving overdiagnosis by nonspecialists and thus increasing the number of food allergens. On the other hand, it has been reported that severe food allergy patients have a tendency to be allergic to many food allergens, comparatively [32]. There is a high possibility that true food allergy students whose food allergies have been determined by properly going through a doctor’s diagnosis are seriously ill and may not have been able to acquire tolerance before the school term began and this may be the cause of the increase in food allergen numbers in the order of Non-Diagnosis group < Single Diagnosis group < Double Diagnosis group. However, further consideration is necessary in the future for confirming this speculation.

The increase in health issues related to diet and food allergy in children is becoming a serious problem in Japan, similarly to that of other countries. Child development is crucially affected by their food intakes, including school lunch, and the risk of food allergies can be hindered by necessary and appropriate accommodations that may ensure that every child is receiving nutritionally-balanced diet. The status of unnecessary accommodations being made to unconfirmed food allergy students per school lunches may draw the attention of the school leadership and managements, teachers, guardians, and lunch service kitchens to the importance of continuous medical documentation such as allergen-specific IgE blood and oral food challenge tests, and communication between school and guardians. In addition, the present study has been able to obtain baseline data to verify the Guideline policy effects notified by the Japanese Ministry of Education, Culture, Sports, Science and Technology in March 2014 [24], of how the percentage of unconfirmed food allergy students changed by urging the submission of a doctor’s diagnosis.

The limits to the present study are, first, the participants surveyed were only the school lunch service kitchens in Okinawa. Second, in order to verify the presence of unconfirmed food allergy students, it would be necessary for the students to receive a diagnosis from a doctor and confirm that they do not have a food allergy. Future issues to be pointed are for consideration are to increase the survey participants and area, and to develop a survey method that would help determine unconfirmed patients. On the other hand, Okinawa Prefecture, who served as survey participants, has approximately the same food allergy support rate and content as the national average [2] and, because of this, it is thought that the present study can be applied as general results for the rest of Japan.

## 5. Conclusions

The present study confirmed that there was information that is medically inconsistent, information suggesting acquisition to tolerance, and information inferring excessive worrying by guardians in Okinawa, Japan, and suggests the possibility that unconfirmed children with food allergies may be receiving unnecessary food allergy accommodations per school lunches. Requiring food allergy tests and a doctor’s diagnosis not only in the beginning of giving food allergy accommodations but also during them are thus very important, as made clear by the lower percentage rates of food allergy students in the school lunch service kitchens with medical documentation at the start and during provisions being made.
